# Impact of the Maternal and Child Health handbook in Angola for improving continuum of care and other maternal and child health indicators: study protocol for a cluster randomised controlled trial

**DOI:** 10.1186/s13063-020-04664-w

**Published:** 2020-08-24

**Authors:** Olukunmi Omobolanle Balogun, Caroline Kaori Tomo, Keiji Mochida, Masashi Mikami, Henda da Rosa Vasconcelos, Isilda Neves, Hisakazu Hiraoka, Hirotsugu Aiga, Rintaro Mori, Kenji Takehara

**Affiliations:** 1 National Center for Child Health and Development, Tokyo, Japan; 2 TA Networking Corp., Tokyo, Japan; 3 Department of Global Health, Graduate School of Health Sciences, University of the Ryukyus, Nishihara, Japan; 4 National Directorate of Public Health, Luanda, Angola; 5 Japan International Cooperation Agency, Tokyo, Japan; 6 School of Tropical Medicine and Global Health, Nagasaki University, Nagasaki, Japan; 7 Graduate School of Medicine, Kyoto University, Kyoto, Japan

**Keywords:** Continuum of care, Service utilisation, MCH handbook, Maternal and child health outcomes, Angola

## Abstract

**Background:**

The Maternal and Child Health (MCH) handbook is an integrated home-based record (HBR) designed to record in a single document all the information regarding health services provided to a pregnant woman and her child. The MCH handbook has the potential to support continuity of care which is key to strengthening maternal, newborn and child health. However, there is a lack of an integrated system to manage the health of pregnant women and young children on an ongoing basis in Angola. Thus, the Angolan Ministry of Health is partnering with the Japan International Cooperation Agency to build the capacity of healthcare providers through trainings and implementation of the MCH handbook to improve service utilisation. In this study, we will estimate the impact of an intervention package including distribution of MCH handbook and its supplementary interventions to women, on the utilisation of services provided at healthcare facilities from pregnancy through the postnatal and early childhood period.

**Methods:**

This study is a cluster randomised controlled trial involving public healthcare facilities across all the municipalities located in Benguela Province, Angola. All women who go to participating healthcare facilities and with confirmed pregnancy around the beginning of the trial period will be included in the study. Women will be randomised according to the municipality where their primary maternity and/or childcare services are located. The intervention package will consist of MCH handbook distribution at all public healthcare facilities, MCH handbook utilisation training for healthcare providers and community mobilisation for women on the use of the MCH handbook. The intervention will be administered to all women in the intervention arm while those in the control arm will continue the traditional use of two stand-alone HBRs. The primary outcome measure for this study is to compare the proportion of women who achieve a complete continuum of care in both study arms.

**Discussion:**

The findings from the study are expected to form a basis for revising the current trial version of the Angola MCH handbook and provide a framework for policy guiding nationwide scale-up and distribution of the MCH handbook.

**Trial registration:**

ISRCTN Registry ISRCTN20510127. Registered on 4 June 2019

## Background

The Republic of Angola, after almost 30 years of civil war, has made substantial economic and political progress in recent years [1], and such changes in the political, social and economic development of Angola directly affect population health. While the development of the health sector has not been universal within the country, the government and its partners are making continuous effort at improving access to health services [2]. The National Health System is today a landmark of a social policy geared towards human development [3], but there is still a long way to go. Health indicators are yet to reach adequate standards, and women and children’s health continues to suffer. Maternal health is an important indicator in determining the status of health in a country and maternal mortality remains high in Angola. According to the World Health Organization (WHO) estimates, approximately 477 maternal death occurred per 100,000 live births in 2015 [4], primarily due to preventable causes. Also, Angola remains one of the African countries with the highest burden of under-5 (81 deaths per 1000 live births) and infant (54 deaths per 1000 live births) mortality rates, despite a consistent decreasing trend in recent years [5]. Some of the factors contributing to the poor maternal and child health indicators in Angola include weak health management systems [6] and the lack of an integrated system to manage the health of pregnant women and young children on an ongoing basis.

Towards rebuilding and strengthening the primary health care in Angola, its Ministry of Health (MoH) with support from the World Bank formally launched the Municipal Health Service Strengthening Project and the campaign for the acceleration of maternal and infant mortality reduction in 2010 [7]. This involves providing universal access to a package of high-impact interventions, including antenatal care (ANC) and counselling for women, provision of insecticide-treated bed nets for pregnant women and children under five to prevent malaria, immunisations against vaccine-preventable childhood diseases, de-worming and delivery of essential micronutrients [1]. Care for women and children starting from pregnancy through childbirth into the babies’ childhood has been promoted to improve maternal and child health through a continuum of care (CoC) [8, 9]. Continuum of care involves the concept of an integrated system of care that guides and tracks mothers and babies over time, through a comprehensive array of health services involving all levels of intensity of care. Continuum of care for mothers, newborn and children forms the basis of health care in many high-income countries which also have the best maternal health indicators [8]. In contrast, health care is neither continuous nor integrated in many low-income countries, including Angola.

The Government of Angola recently developed the National Plan for Health Development 2012–2025 and prioritised pregnant women, infants and young children as the target population. With support from the United Nations (UN), Angola is also working to strengthen the health information system in the collection, processing and analysis of data [6]. A mechanism for attaining this goal involves an emphasis on strengthening institutional and management capacity, and capacity building for health workers through long- or short-term trainings. Given the political will, the Japan International Cooperation Agency (JICA), through its health sector cooperation, is providing support to the Government of Angola to enhance healthcare service delivery. Moreover, JICA is also supporting the MoH through its Technical Cooperation Project (TCP) “Project for Improving Maternal and Child Health Services through the implementation of the Maternal and Child Health (MCH) handbook”.

The MCH handbook is an integrated home-based record (HBR) and is part of a scheme designed to record, in a single document, all the information and data regarding health services provided to and the health conditions of a mother and her child during the course of pregnancy, delivery and after birth, such as maternal care and the child’s growth pattern and vaccination schedule [10, 11]. In addition to other uses, the MCH handbook functions as a self-learning material, has the potential to reduce the need for multiple health records [12] and supports improvements in CoC [13, 14]. As a result, the MCH handbook has been attracting more attention from health ministries and professional organisations as an effective tool for promoting a life course approach to health care [11].

Despite the potentially important benefits of implementing the MCH handbook, there is a dearth of high-quality studies to show its superiority to existing alternatives [15, 16]. Therefore, this study will estimate the impact of an intervention package including distribution of MCH handbook and its supplementary interventions to women, compared with the traditional use of two stand-alone HBRs the on utilisation of services provided at healthcare facilities from pregnancy through the postnatal and early childhood period.

## Methods

### Study objectives and design

This study is a two-arm cluster randomised controlled trial (cRCT) to estimate the impact of a community-wide distribution of the MCH handbook and its supplementary interventions to women, compared with the traditional use of two stand-alone HBRs on CoC completion and various MCH outcomes. The unit of randomisation in this trial is the municipality (cluster). Specifically, this study will (1) evaluate the impact of MCH handbook on CoC completion, (2) assess uptake and utilisation of MCH handbook by both families and healthcare providers in Angola and (3) assess the impact of MCH handbook distribution and utilisation on maternal and child health.

### Study site

The study will be carried out in Benguela Province, Angola. Benguela is situated in the west of the country. Benguela has 10 administrative divisions called municipalities and a population of approximately 2.6 million [17]—being the third most populous province in Angola. Its population is mostly ethnic Ovimbundu and the most widely spoken language besides Portuguese is Umbundo. The most populous municipalities in the province are Lobito, Benguela, Cubal and Ganda (in descending order) [17]. Agriculture, fisheries and livestock farming are the main economic activities of this population. Benguela was purposively selected for this study because of health indicators that are close to the national average, thus representing an ideal setting to show intervention impact on MCH outcomes and demonstrate feasibility for nationwide scaling up of MCH handbook in Angola.

### Cluster selection

This impact evaluation study will be conducted in all 10 municipalities of Benguela Province. Each municipality comprises health communes that form the health administrative units for the province. The number and level of care provided by healthcare facilities vary between the municipalities. There are currently 209 public healthcare facilities [18] serving health needs and providing maternal newborn and child health (MNCH) services across Benguela Province comprising 82 secondary- or tertiary-level hospitals and 127 primary healthcare units (Table 1). The primary healthcare level provides services including MNCH care and refers cases to secondary and tertiary healthcare level as needed.

**Table 1:** Number of healthcare facilities in Benguela Province

Arm	Municipality	Level of care		Total
		Primary	Secondary/tertiary	
Intervention	Lobito	24	3	99
	Cubal	20	2	
	Chongoroi	16	1	
	Bocoio	17	1	
	Balombo	15	0	
Control	Benguela	30	5	106
	Catumbela	16	1	
	Baia Farta	15	0	
	Ganda	24	3	
	Caibambo	11	1	
Total		188	17	205

There were 116,032 pregnant women in Benguela Province with 95,950 registered births in 2015. Of these, about 38% of women attended ANC services at a healthcare facility ≥ 4 times [18]. Furthermore, ANC attendance varied by municipality whereby ANC service utilisation was highest in Benguela (60%), Chongoroi (48%) and Lobito (47%) and lowest in Balombo (15%) and Baia Farta (4%) in 2015. In the same year, the rate of facility-based deliveries in Benguela Province was limited to 45%, while 93% of children in the province were fully immunised [18]. Various factors, such as distance to the healthcare facility, affect women’s utilisation of MNCH services across the province [19].

### Participant cohort

**Fig. 1:**
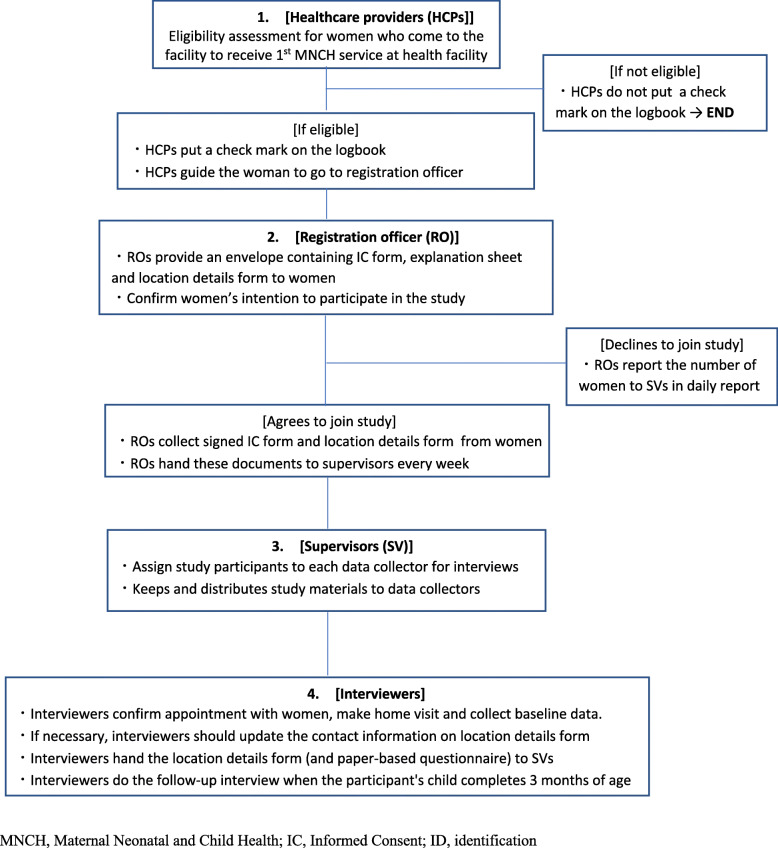
Participant recruitment process and data collection team tasks

The intervention will be administered to pregnant women through frontline providers of MNCH services in public healthcare facilities in Benguela. The impact of the intervention will be estimated by collecting data about maternal and newborn participants who seek care from participating facilities. All women pregnant at the beginning of the trial period and attending a public healthcare facility for MNCH services will be eligible for inclusion in the study. In order to ensure similar baseline characteristics among participant cohort across all municipalities, the study cohort will include all women confirmed to have become pregnant at the start of the trial implementation period. The participant cohort will be recruited at their first visit for any MNCH service consultation over the course of the study **(**Fig. 1). Based on the findings from a previous study [20], most pregnant women are expected to make their first visit to the healthcare facilities during the second trimester of pregnancy.

**Fig. 2:**
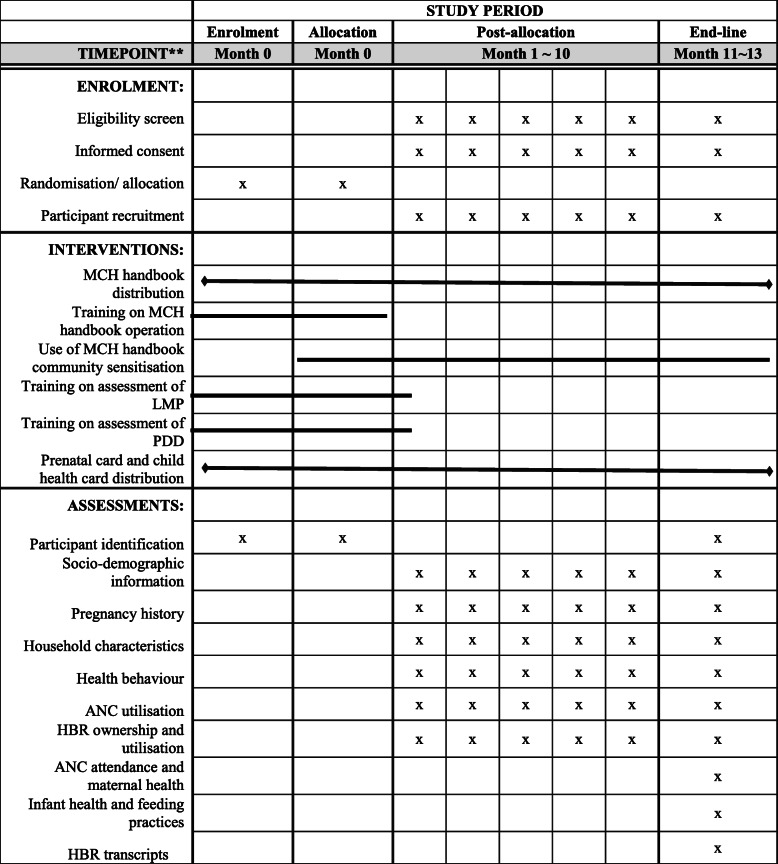
schedule showing enrolment, allocation, intervention, baseline and end-line assessments. MCH, maternal and child health; LMP, last menstrual period; PDD, probable delivery date; ANC, antenatal care; HBR, home-based record

**Fig. 3:**
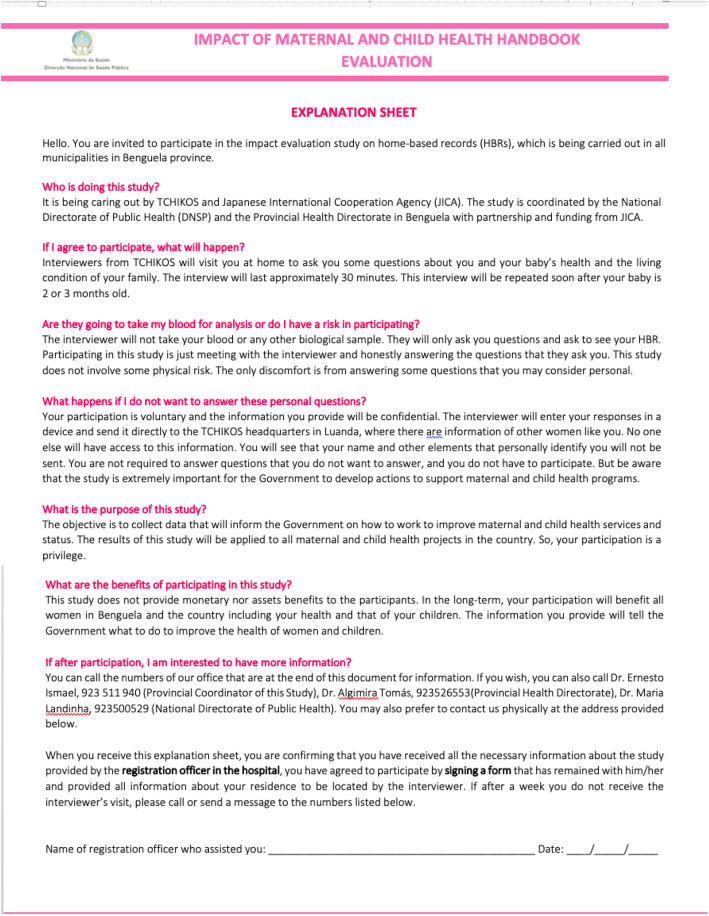
Participant information sheet

**Fig. 4:**
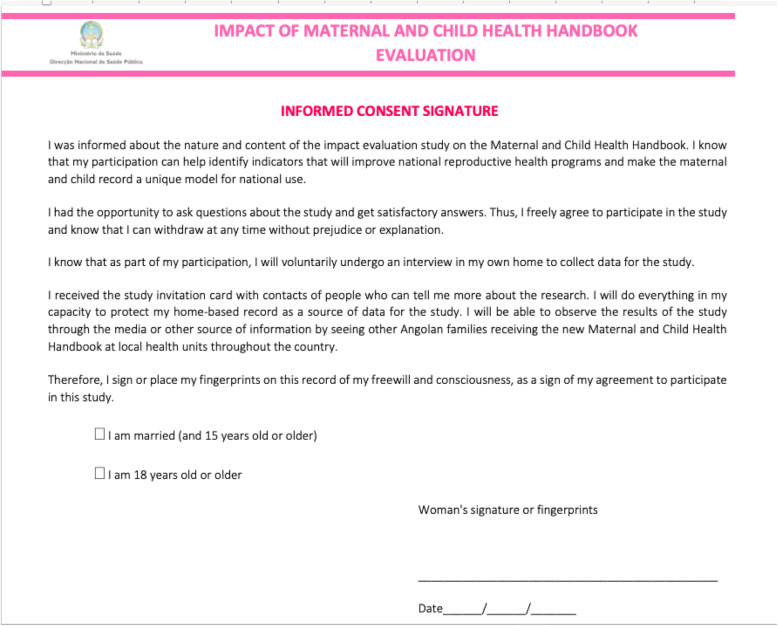
Participant informed consent form

Prior to recruitment, study eligibility will be assessed and pregnant women who satisfy these criteria will be briefed about the trial and invited to participate. Consenting participants will then be registered and enrolled in the study. Figure 2 shows the study timeline according to the Standard Protocol Items: Recommendations for Interventional Trials (SPIRIT) diagram. For each registered participant, contact details will be collected in as many details as possible to enable follow-up home visits by the trial team. Written informed consent will be obtained from every participant joining the study prior to enrolment. Further, all participants will be assured of their rights to voluntary participation, privacy, confidentiality and free choice to withdraw from the study during the conduct of the trial in the process of obtaining informed consent (Fig. 3 in Appendix 1, Fig. 4 in Appendix 2).

### Eligibility criteria

Participants will be eligible to join the study if they are confirmed by a healthcare provider to have become pregnant at the beginning of the trial period, had her last menstrual period (LMP) on any date from 1 March to 30 April 2019 or probable delivery date (PDD) on any date between 1 December 2019 and 31 January 2020. Participants will be selected among pregnant women and mothers within the defined cohort on their first visit to public healthcare facilities for MNCH services. Pregnant women will be excluded if they decline to participate in the study or are planning to move out of the study area while the impact evaluation study is ongoing.

### Power analysis

This cRCT has three unit levels: the municipality, healthcare facility and the individual. A simulation-based power analysis was done in SAS software 9.4 (SAS Inc., Cary, NC, USA) using the generalised linear mixed model and taking these levels into consideration (Table 6 in Appendix 3). The power analysis was used to estimate the sample size needed to detect a 10% (25% in the intervention arm and 15% in the control arm) increase in the rate of CoC completion significant at 5% and assuming a 20% dropout rate. The intracluster correlation coefficients (ICC) used in this simulation-based power analysis were 0.010, 0.015 and 0.020 [21]; and assuming that the number of participants was 5000, 7500 and 10,000, the power ranged from 0.560 to 0.828. We also confirmed in the simulation that type 1 error does not inflate. On the basis of these calculations, we aim to recruit at least 10,000 participants. The target sample size in each municipality will vary depending on the population size.

### Randomisation and allocation concealment

This study is a two-arm study comprising an intervention arm and a control arm. The unit of randomisation in this trial is the municipality (cluster). Cluster randomisation was chosen due to the nature of the intervention and to minimise logistical and scientific difficulties associated with individual randomisation in large-scale longitudinal studies—such as contamination. Benguela Province consists of 10 municipalities of varying sizes—Baia Farta, Balombo, Benguela, Bocoio, Caimbambo, Catumbela, Chongoroi, Cubal, Ganda and Lobito. All 10 municipalities will be included in the study. Population size and basic characteristics of each of the 10 municipalities were taken into consideration during the randomisation process to ensure a balance of socio-demographic characteristics between both arms.

Municipalities were allocated to either intervention or control arms using block randomisation [22, 23] by an experienced statistician in two steps. To ensure a balance between the two study arms, we ordered the municipalities by population size then applied block randomisation as described by Gattellari et al. [24]. First, municipalities were sorted by size based on the 2014 population data provided by the National Institute of Statistics [25]. Using the data, five blocks comprising of two municipalities each were created in the order of population size. Second, municipalities were randomly allocated either to the intervention or to the control arm within each block using computer-generated results. In this step, a pair of 4-digit code assigned to each block was fed into a SAS program that automatically returned a response, assigning one of the municipalities in the block to the intervention or control arm. The statistician was unable to predict assignment to either study arm. Municipalities allocated to the intervention or control arms were revealed simultaneously to the study team and municipal authorities at the kick-off meeting marking the start of the trial.

### Blinding (masking)

Blinding will not be feasible due to the nature of the intervention; thus, neither participants nor trial personnel will be blinded in this study. However, the data analyst will be blinded to the allocation.

### Intervention

The Angolan MCH handbook is a 36-page HBR designed to keep each pregnant woman’s medical record from pregnancy through early childhood with educational information. It was developed based on the prenatal card and child health card currently being used in Angola, UNICEF educational materials, MoH documents and other materials [26]. The MCH handbook contains a log for making entries on maternal personal and health information during the course of pregnancy, delivery and postpartum; weight during and after pregnancy; infectious disease prevention and control; infant nutrition; child developmental milestones from the ages of 0–59 months; vaccination and illnesses records; and growth charts for children. The cultural appropriateness and acceptability of the handbook among pregnant women and healthcare providers have been previously assessed within a small cohort [27]. Even though the MCH handbook was generally well received among both mothers and healthcare providers, training for both groups would be required to derive the full benefits of the MCH handbook. Thus, the intervention consists of three basic components: (i) distribution of MCH handbook (the MCH handbook will be issued to pregnant women at each health facility at the HBR distribution points in the intervention arm), (ii) healthcare provider training on MCH handbook operation and (iii) community sensitisation and mobilisation among pregnant women on the use of the MCH handbook. Interventions will be delivered to pregnant women and healthcare providers in the intervention arm.

#### Pregnant women/mothers

The MCH handbook will be provided to the participant cohort during their first visit to receive MNCH services in each participating healthcare facility in the intervention arm. Explanation will be given on when and how to use the MCH handbook and on data recording. Participants will also receive health education based on the contents of the MCH handbook. Pregnant women and mothers in the study cohort attending healthcare facilities in control municipalities will continue to benefit from the traditional use of two stand-alone HBRs—the prenatal card and child health card. Pregnant women and mothers in both intervention and control municipalities will be asked to bring their MCH handbook or prenatal card and child health card to every occasion of MNCH service consultation. If a participant in either arm forgot to bring her handbook/card during a consultation, the attending healthcare provider will make a copy of the relevant recording page which will be used for keeping the record of the consultation and given to the participant.

#### Healthcare providers

Healthcare provider training on MCH handbook operation will be provided prior to distribution of the MCH handbook to pregnant women/mothers. They will be trained on skills needed for data recording into the MCH handbook, how to explain the use of the MCH handbook to pregnant women/mothers, make entries into the MCH handbook during regular consultations with pregnant women/mothers and on how to interpret data entries from the MCH handbook. Healthcare providers in the control arm will be trained on interviewing techniques for determining the date of LMP and on methods for calculating PDD only (Table 2). Healthcare providers in both intervention arms will be re-trained periodically over the intervention period.

**Table 2:** Description of interventions

Description	Intervention arm	Control arm
Distribution of MCH handbook	x	
ToT on MCH handbook operation	x	
OJT on MCH handbook operation	x	
Assessment of LMP	x	x
Determining PDD	x	x
Community sensitisation and mobilisation on the use of the MCH handbook	x	

*ToT* training of trainers (trainers included representatives from each healthcare facility in the intervention arm), *OJT* on-the-job training for other healthcare providers, *LMP* last menstrual period, *PDD* probable delivery date

### Data collection

Study data collectors will collect data regarding MNCH services participants received. All data will be collected using a structured questionnaire by interviewer-facilitated tablet-based face-to-face interviews or abstracted from HBRs (MCH handbook in intervention arm or prenatal card and child health card in the control arm). However, where there is no record of MNCH services received in HBRs, mother’s report of service utilisation will be used in place of the missing information as previously reported by Croft et al. [28]. Data collection will be conducted at participants’ home or at a location of her choice at two time points—at baseline and at 3 months postpartum. Table 3 shows the data collection timeline for each outcome measure.

**Table 3:** Data collection timeline for each outcome measure

Time point	Item	Data source		Location
		Survey form	HBR	
Baseline	Participant identification	x		Healthcare facility
	Socio-demographic information	x		Participants home
	Pregnancy history	x	x	
	Household characteristics	x		
	Health behaviour	x		
	ANC utilisation	x	x	
	HBR ownership and utilisation	x	x	
Follow-up (3 months postpartum)	Participant identification	x		Participants home
	HBR ownership and utilisation	x	x	
	Health behaviour	x		
	ANC attendance and maternal health	x	x	
	Infant health and feeding practices	x		
	HBR transcripts		x	

*HBR* home-based record, *ANC* antenatal care

The baseline survey will comprise eight sections designed to elicit responses on socio-demographic factors, household characteristics, pregnancy history, maternal health behaviour and HBR ownership and utilisation. Data regarding ANC attendance, maternal health behaviour, pregnancy outcomes, infant feeding practices and infant and maternal health will also be collected from women retrospectively during the follow-up survey at 3 months postpartum. Verbal responses obtained from mothers will be double-checked against record entries made in the HBRs. In cases where discrepancies occur between the mother’s report and HBR records, the latter will be prioritised. Additionally, we will verify the quality of HBR records by referring to the facility logbook data collected from a randomly selected sub-sample of women.

Interviews and data abstraction will be carried out by data collectors. Data collectors were selected from among persons with medical training or experience working with patients and were trained in tablet-based questionnaire administration and data collection processes. Training of the data collectors consisted of an overview of the study objectives, detailed description of baseline and follow-up questionnaire items and interviewing techniques, and field practice. The baseline and follow-up questionnaires were pre-tested before the start of the study. A summary of data collection timeline and questionnaire items is shown in Table 3. Interview responses and data abstracted by each data collector will be uploaded to a secure database and stored in dedicated computers on an on-going basis. Verification checks will be done on uploaded records, and attempts will be made to ensure the completeness and accuracy of collected data.

### Outcome measures

The primary outcome for this study is a measure MNCH service utilisation assessed via the proportion of women who achieved time-dependent maternal behaviour-based CoC completion as at 3 months postpartum. MNCH service utilisation and CoC completion among women in low- and middle-income countries have been found to be associated with many factors such traditional and familial beliefs, distance to healthcare facility [29, 30], health care cost [29–31], maternal education [30], perceived quality of care [29, 31] and service availability [32]. Therefore, factors influencing CoC completion may depend either on maternal behaviour and characteristics (demand-side factors) or service availability (supply-side factors). In this study, we hypothesise that distribution and utilisation of the MCH handbook will lead to maternal behaviour change leading to improved health-seeking behaviour and MCH outcomes. Consequently, the primary outcome is a time-dependent maternal behaviour-based measure of CoC completion. CoC completion will be assessed among all pregnant women who were recruited to the study during their ANC attendance. Under this measure, a time-dependent service utilisation-based CoC completion will be achieved when pregnant women receive MNCH services including a minimum number of ANC visits, facility-based delivery, postnatal care (PNC) for mother and newborn, and child vaccinations at two time points—at birth and at 2~3 months of infant age. MNCH service utilisation assessed via the proportion of women who achieve time-dependent service-based CoC completion as at 3 months postpartum will be assessed as a secondary outcome. Coverage under this measure would depend on supply-side factors, such as service availability. Thus, CoC completion will be achieved only when pregnant women/mothers have received all the required MNCH services as shown in Table 4. Details of other secondary outcomes to be assessed are presented in Table 4.

**Table 4:** List of outcomes and definition of outcome indicators

Outcome	Definition	Type	Indicators	Details
**Primary outcome**				
Complete CoC (maternal behaviour-based)	Time-dependent composite outcome. Complete CoC is the minimum number of ANC visits + facility-based delivery + PNC for mother and newborn + child vaccinations at two time points—at birth and at 2~3 months. Includes women who attended ANC and other MCNH services only. Complete CoC = 1 when all indicators = 1	Binary	First ANC timing	Timeline divided into ≤ 20, 21~30, 31~37 and 38 weeks~ ga
			No. of ANC services	< 4 = 0, ≥ 4 = 1 (≤ 20 weeks); < 3 = 0, ≥ 3 = 1 (21–30 weeks); < 2 = 0, ≥ 2 = 1 (31~37 weeks); < 1 = 0, ≥ 1 = 1 (38 weeks)
			Facility delivery	No = 0; yes = 1
			PNC_mother	No HBR record = 0; HBR record = 1
			PNC_child	No HBR record = 0; HBR record = 1
			Vaccinations at birth and at 2 months infant age	< 2 vacc clinic visits = 0; 2 vacc visits = 1
**Secondary outcomes**				
Rate of MNCH service utilisation	Total no. of actual MCH service used per person per arm compared with an expected minimum number of service utilisation. Estimate means for each arm	Continuous	No. of ANC services	No. of women with a minimum number of ANC in each arm
			Facility delivery	No of women with facility delivery in each arm
			PNC_mother	No. of women with mother PNC record in each arm
			PNC_child	No. of women with child PNC record in each arm
			Vaccination	No. of women with child vacc record in each arm
Complete CoC (service-based)	Time-dependent composite outcome. Composite outcome including ≥ 4 ANC visits + facility-based delivery + PNC of mother and newborn + complete child immunisation up to 2~3 months. Complete CoC = 1 when all indicators = 1	Binary	First ANC timing	Timeline divided into ≤ 20, 21~30, 31~37 and 38 weeks~ ga
			No. of ANC services	< 4 = 0, ≥ 4 = 1 (≤ 20 weeks); < 3 = 0, ≥ 3 = 1 (21–30 weeks); < 2 = 0, ≥ 2 = 1 (31~37 weeks); < 1 = 0, ≥ 1 = 1 (38 weeks)
			Facility delivery	No = 0; yes = 1
			PNC_mother	No HBR record = 0; HBR record = 1
			PNC_child	No HBR record = 0; HBR record = 1
			Vaccination	< 7 records = 0; 7 records = 1
Neonatal mortality	No. of deaths within the first 28 days of life compared to the total number of deliveries in each study arm. NM = 1 when all indicators = 1	Binary	Live birth	No = 0; yes = 1
			Infant death	No = 0; yes = 1
			Date of death	Death within 28 days of birth = 1
ANC service utilisation	No. of women who receive ≥ 4 ANC visits in each arm regardless of the timing of first ANC. Adequate ANC = 1 if no. of ANC services = 1	Binary	First ANC timing	1st trimester, 2nd trimester, 3rd trimester
			Received ANC	No = 0; yes = 1
			No. of ANC services	< 4 = 0; ≥ 4 = 1
	No. of women who receive ≥ 8 ANC visits in each arm regardless of the timing of first ANC. Adequate ANC = 1 if no. of ANC services = 1	Binary	First ANC timing	1st trimester, 2nd trimester, 3rd trimester
			Received ANC	No = 0; yes = 1
			No. of ANC services	< 8 = 0; ≥ 8 = 1
Facility-based delivery	No. of facility-based deliveries in each arm.	Binary	Facility delivery	No = 0; yes = 1
Infant health check-up	Presence of at least one HBR entry from baby-well clinic attendance.	Binary	Record entry for any of current wgt, polio1, pentavalent, pneumo1 or rota1	No HBR record = 0; HBR record (for any of current wgt, polio1, pentavalent, pneumo1 or rota1) = 1
Maternal morbidity and pregnancy complication detection rate	No. of cases of specified disease conditions and pregnancy complications diagnosed by an HCP in each arm. Maternal morbidity = 1 if any indicator = 1	Binary	High blood pressure	No HBR record = 0; HBR record = 1
			Preeclampsia	No HBR record = 0; HBR record = 1
			Miscarriage	No HBR record = 0; HBR record = 1
			Stillbirth	No HBR record = 0; HBR record = 1
			Vaginal bleeding	No HBR record = 0; HBR record = 1
			Anaemia	No HBR record = 0; HBR record = 1
			Malaria	No HBR record = 0; HBR record = 1
			TB	No HBR record = 0; HBR record = 1
			HIV	No HBR record = 0; HBR record = 1
			Diabetes	No HBR record = 0; HBR record = 1
Infant morbidity rate	Total no. of disease cases attended by an HCP. Infant morbidity = 1 if all indicators = 1	Binary	Past month sickness	No = 0; yes = 1
			Sought treatment	No = 0; yes = 1
			Treatment from HP	No = 0; yes = 1
Infant mortality	No of deaths of infants < 1 year old compared to the total number of deliveries in each study arm. IM = 1 when if both indicators = 1	Binary	Livebirth	No = 0; yes = 1
			Infant death	No = 0; yes = 1
Maternal health behaviour	Prevalence of maternal health behaviour between arms with regard to tobacco use, alcohol use, PMTCT, malaria prevention, use of ITNs and family planning. Prevalence of each indicator will be compared between the intervention and control arms.	Binary	Current alcohol use	No = 0; yes = 1
			Current tobacco use	No = 0; yes = 1
			PMTCT	No = 0; yes = 1
			Family planning use	No = 0; yes = 1
			Knowledge change tobacco use	No = 0; yes = 1
			Knowledge change alcohol use	No = 0; yes = 1
Malaria prevention		Binary	ITN possession	No = 0; yes = 1
			ITN use	No = 0; yes = 1
			Malaria prophylaxis use	No = 0; yes = 1
Maternal depression	No. of cases of maternal postnatal depressive symptoms in each arm.	Binary	EPDS	< 10 = 0; ≥ 10 = 1
Infant feeding practices	Appropriate infant feeding practices including EIBF, EBF and absence of pre-lacteal feeding. Adequate if all indicators = 1	Binary	EIBF	After 1 h = 0; within 1 h = 1
			Pre-lacteal feeding	Yes = 0; no = 1
			Current BF	No = 0; yes = 1
Child vaccination	Composite outcome for the no. of complete fully vaccinated children at 3 months in each study arm. Vaccination = 1 if all indicators = 1	Binary	Polio0	No HBR record = 0; HBR record = 1
			BCG	No HBR record = 0; HBR record = 1
			Hepatitis B	N HBR record = 0; HBR record = 1
			Polio1	No HBR record = 0; HBR record = 1
			Pentavalent	No HBR record = 0; HBR record = 1
			Pneumo1	No HBR record = 0; HBR record = 1
			Rotavirus1	No HBR record = 0; HBR record = 1

*CoC* continuum of care, *ANC* antenatal care, *PNC* postnatal care, *HBR* home-based record, *MNCH* maternal, newborn and child health, *NM* neonatal mortality, *IM* infant mortality, *PMTCT* prevention of mother-to-child transmission, *ITN* insecticide-treated net, *EIBF* early initiation of breastfeeding, *EBF* exclusive breastfeeding

### Other secondary outcomes

1. Rate of MNCH service utilisation—number of actual MNCH service used
2. Neonatal mortality—number of infant deaths within the first 28 days of life
3. ANC service utilisation—frequency of ANC service use in each arm
4. Facility-based delivery—number of facility-based deliveries in each arm
5. Infant health check-up—proportion of children who received infant health checks
6. Maternal morbidity and pregnancy complication detection rate—number of cases of specified disease conditions and pregnancy complications diagnosed by a health care provider
7. Infant morbidity rate—number of specific childhood disease cases attended by a health care provider
8. Infant mortality—number of infant deaths in each study arm
9. Maternal health behaviour—prevalence of maternal health behaviour between arms measured using at baseline and follow-up
10. Maternal depression—number of cases of maternal postnatal depressive symptoms
11. Infant feeding practices—prevalence of appropriate infant feeding practices in each study arm
12. Child vaccination—number of fully vaccinated children at the end of the study period

All outcomes will be assessed at follow-up except otherwise stated, and data will be collected through structured interviews and abstracted from HBRs.

The minimum number of MNCH services required for maternal behaviour-based CoC completion is shown in Table 5 and will depend on the timing of the first ANC visit and exposure to the intervention.

**Table 5:** Minimum expected number of service utilisation to achieve complete CoC (primary outcome)

Time of first ANC (weeks)	ANC	Del	PNCm	PNCc	Vacc	Total	Complete CoC
≤ 20	4	1	1	1	2	9	4 ANC, del., PNCm, PNCc, 2 vacc visits
21~30	3	1	1	1	2	8	3 ANC, del., PNCm, PNCc, 2 vacc visits
31~37	2	1	1	1	2	7	2 ANC, del., PNCm, PNCc, 2 vacc visits
38~	1	1	1	1	2	6	1 ANC, del., PNCm, PNCc, 2 vacc visits

*ANC* antenatal care, *del.* facility delivery, *PNCm* postnatal care for mother, *PNCc* postnatal care for child, *vacc* two vaccination clinic visits at birth and 2 months infant age

### Statistical analysis

The intervention arm (MCH handbook) will be compared to the control (traditional use of two stand-alone HBRs) in the main analysis. We will conduct descriptive analysis, and baseline data from the intervention and control arms will be analysed for comparability of baseline characteristics. Rates of missing data for each variable will be determined to assess the differences between the arms, and overall losses to follow-up will be determined. We will report reasons for losses within each arm and compare the reasons qualitatively.

We plan to measure intervention impact using two analysis sets: the intention-to-treat (ITT) set and the per-protocol analysis set (PPS). The ITT population will include all participants within the randomised municipalities analysed according to the municipality where they were first registered to receive MNCH services (i.e. discounting where they received second, third or fourth MNCH services) [33, 34]. The ITT population will be the primary analysis set, and all analyses will be conducted using this population unless otherwise stated. In the PPS, participant data will be analysed according to the treatment they actually received. Data from the participant with more than one MCNH service utilisation in the control arm will be included in the control arm regardless of the municipality of first registration for MNCH services in the PPS analysis.

The primary endpoint in this impact evaluation study is to compare the proportion of CoC completion achieved among mothers who received the MCH handbook in the intervention arm and those who received the traditional stand-alone HBRs in the control arm. We will evaluate the effect of the provision of MCH handbooks on CoC completion at the municipality level using a generalised linear mixed model (GLMM) assuming logit link and binomial distribution. All analyses will be based on complete case analysis. Regarding the number of healthcare service utilisations, the analysis will be conducted using survival analysis. We will also evaluate the intervention effects in a subgroup of participants based on socio-economic status, urban/rural residence and other baseline characteristics while accounting for cluster effect.

All analyses will be done using STATA (version 13 or higher, Stata Press, College Station, TX).

### Losses to follow-up

Participants who do not provide any baseline or follow-up data and those who withdraw their consent to participate in the study will be regarded as being lost to follow-up. To minimise such losses, a participant identification form will be used to collect each woman’s contact information in as much detail as possible, including GPS location where baseline interview was conducted to facilitate repeat contact with mothers during the follow-up survey. The proportion of participants lost to follow-up will be compared between the two arms.

### Data management and storage

All data collection processes will be standardised across the 10 municipalities. At each healthcare facility, pregnant women seeking MNCH services and eligible to join the study will be listed in the study register maintained by a data collector. Following enrolment, each woman will be given a unique identification number. A standardised tablet-based data collection form will be administered to each woman by trained study data collectors at baseline and follow-up. To ensure completeness of each record, virtually, all questions in the form are required, while at the same time ensuring participants right to voluntary participation. Completed forms will be uploaded to a secure server at the end of each day. The datasets will be password-protected and only accessible to the data manager.

We will not establish a third-party data monitoring committee due to the nature of the intervention and study design. However, periodic meetings involving all members of the study team will be held weekly to review the processes and progress related to the following: (1) participant recruitment and interviews; (2) completeness, accuracy and timeliness of uploading completed survey forms to secure server; and (3) any other logistical issues related to the study.

### Risk and benefit

The intervention in this study is non-invasive and carries a less than minimal risk for adverse events in participating women. Consequently, potentially serious outcomes are not anticipated, and we do not envisage any reason, due to the intervention or study design to end the trial prematurely. Therefore, we will not conduct an interim analysis. Notwithstanding, disclosure of personal information and time taken for survey interviews may cause some discomfort to some participants. Data collectors will assure each participant of the anonymity and confidentiality of the data collected to make participants at ease. Adverse events that may arise will be assessed by the study team and reported to the responsible ethics committee. Reporting will include the type of event, circumstances, the extent of connection with study activities and the response provided. Potential benefits to participants in the study include improved CoC completion, knowledge of recommended health practices during pregnancy, recognition of danger signs and care of mother and newborn. Moreover, the intervention package will be delivered through the existing healthcare system for obtaining MNCH services.

## Discussion

This is a study protocol for a cRCT aimed at estimating the impact of an intervention package—including the distribution of MCH handbook and its supplementary interventions to pregnant women, compared with the traditional use of two stand-alone HBRs on the utilisation of services provided at healthcare facilities from pregnancy through the postnatal and early childhood period. Few studies have shown that proper utilisation of HBRs could promote continuity of maternal and child health care [14, 16, 35], and WHO recommends that each pregnant woman carries her own HBRs during pregnancy to improve continuity and quality of care [36]. Notwithstanding, the superiority of the integrated MCH handbook over other alternatives is an area of research in which there is a dearth of gold standard trials [15, 37]. We will attempt through this trial to provide higher quality evidence to demonstrate the benefits of implementing the MCH handbook over traditional stand-alone HBRs.

As a cluster-level intervention package, the trial employs a population-based approach to ensure uniform distribution of the MCH handbooks and its supplementary interventions. A cRCT is the preferred design in this trial to minimise logistical and scientific difficulties such as contamination between clusters and to optimise conditions to bring the intervention to scale within Angola in a real-life context. The trial’s efficiencies lie in its design and data collection methods. The intervention package will be administered through the existing healthcare system for delivering MNCH services and with the support from the regional and local health divisions who routinely supervise the activities of frontline healthcare providers. Data will be collected through tablet-based interview methods. Mobile tablet devices have become an important tool for collecting primary data in clinical research, using a combination of functions including data entry, audio recorders, cameras, barcode readers and global positioning system receivers [38]. Using this method, the data management team in this trial will more easily collect and link data from the baseline and follow-up surveys. Mobile phones have also become an important tool for follow-up in public health research, through calls and text messaging [39, 40]. In order to reduce attrition rates, we will ask each participant to complete a participant identification form providing information to facilitate repeat contact [39, 41].

The findings from the impact evaluation study will be shared with the Angolan government and are expected to form a basis for revising the current trial version of the MCH handbook and for making an informed policy decision on nationwide scale-up and distribution of the MCH handbook. We plan to disseminate study findings through conference presentations and publication in international peer-reviewed journals.

This study protocol has been reported in accordance with the SPIRIT guidelines (Additional file 1) and including all items from the World Health Organization Trial Registration Data Set (Additional file 2).

### Trial status

This article is the first protocol version. Participant recruitment started in June 2019 and is expected to be completed in June 2020.

### Supplementary information

**Additional file 1.** SPIRIT Checklist: Recommended items to address in a clinical trial protocol and related documents.

**Additional file 2.** Items from the World Health Organization Trial Registration Data Set.

## Data Availability

There are no data available in this article as no datasets were generated or analysed during the study protocol. The full datasets will be available (upon request) after they have been analysed and published.

## Appendix 3

**Table 6:** Sample size simulation

Power		Intervention, 25%; control, 15%		
Simulation: 1000 times *α*=0.05, two-sided		Total *N*		
		5000	7500	10,000
ICC	0.010	0.776	0.808	0.828
	0.015	0.639	0.658	0.674
	0.020	0.560	0.570	0.605

## Abbreviations

ANC: Antenatal care
CoC: Continuum of care
cRCT: Cluster randomised controlled trial
DNSP: National Public Health Directorate
DSP: Directorate of Public Health
ICC: Intracluster correlation coefficient
ITT: Intention-to-treat
ISRCTN: International Standard Randomised Controlled Trials Number
JICA: Japan International Cooperation Agency
LMP: Last menstrual period
MCH: Maternal and Child Health
MoH: Ministry of Health
MNCH: Maternal newborn and child health
PDD: Probable delivery date
PNC: Postnatal care
PPS: Per-protocol analysis set
TCP: Technical Cooperation Project
UN: United Nations
UNICEF: United Nations Children’s Fund
WHO: World Health Organization
