# Supplementary material for: Impact of the Maternal and Child Health handbook in Angola for improving continuum of care and other maternal and child health indicators: study protocol for a cluster randomised controlled trial
Source: Trials. 2020 Aug 24;21:737. doi: 10.1186/s13063-020-04664-w (PMC7445894; doi:10.1186/s13063-020-04664-w)
Supplement: Supplementary file 2 — Additional file 2. Items from the World Health Organization Trial Registration Data Set. [file 13063_2020_4664_MOESM2_ESM.docx]

**Additional file 2:** Items from the World Health Organization Trial Registration Data Set

| Item no. | Data category | Information |
| --- | --- | --- |
| 1 | Primary registry and trial identifying number | ISRCTN Registry ISRCTN20510127 |
| 2 | Date of registration in primary registry | 4 June, 2019 |
| 3 | Secondary identifying numbers | https://doi.org/10.1186/ISRCTN20510127 |
| 4 | Source(s) of monetary or material support | Japan International Cooperation Agency |
| 5 | Primary sponsor | Japan International Cooperation Agency |
| 6 | Secondary sponsor(s) | None |
| 7 | Contact for public queries | Kenji Takehara email: takehara-k@ncchd.go.jp |
| 8 | Contact for scientific queries | Kenji Takehara, National Center for Child Health and Development, Tokyo Japan email:takehara-k@ncchd.go.jp |
| 9 | Public title | Impact of the Maternal and Child Health Handbook in Angola for improving Continuum of Care and other Maternal and Child Health indicators: study protocol for a cluster randomised controlled trial |
| 10 | Scientific title | Impact of the Maternal and Child Health Handbook in Angola for improving Continuum of Care and other Maternal and Child Health indicators: study protocol for a cluster randomised controlled trial |
| 11 | Countries of recruitment | Angola |
| 12 | Health condition(s) or problem(s) studied | MCH handbook distribution, maternal and child health, continuum of care |
|  | Intervention(s) | Intervention: MCH handbook distribution and associated trainings |
| 13 |  | Control: Traditional use of two standalone HBRs |
|  | Key inclusion and exclusion criteria | All women in the study location who become pregnant at the beginning of the trial period |
| 14 |  | Women who decline to participate in the study or are planning to move out of the study area while the trial is ongoing |
|  | Study type | Interventional |
|  |  | Allocation: randomised intervention model; Randomisation unit: cluster; Masking: none |
| 15 |  | Primary purpose: behaviour modification |
| 16 | Date of first enrolment | 8 June, 2019 |
| 17 | Target sample size | 10,000 |
| 18 | Recruitment status | Ongoing |
|  | Primary outcome(s) | Complete CoC (maternal behaviour-based) |
|  |  | Method of measurement: Questionnaire interviews and HBR data retrieval |
| 19 |  | Timepoint: 3 months postpartum (3 months infant age) |
| 20 | Key secondary outcomes | Rate of MNCH service utilisation; Complete CoC (service-based), Neonatal mortality, ANC service utilisation, Facility-based delivery, Infant health check-up, Maternal morbidity and pregnancy complications detection rate, Infant morbidity rate, Infant mortality, Maternal health behaviour, Maternal depression, Infant feeding practices, Child vaccination |
|  |  | Method of measurement: Questionnaire interviews and HBR data retrieval |
|  |  | Timepoint: 3 months postpartum (3 months infant age) |
| 21 | Ethics review | Status: Approved |
|  |  | Date of approval: 1 February, 2018; 12 April, 2018 |
|  |  | Name and contact details: (1 )Ethics Committee of the National Center for Child Health and Development; (2) Ethics Committee for the Ministry of Health, Republic of Angola |
| 22 | Completion date | June, 2020 |
| 23 | Summary results | Not available |
| 24 | IPD sharing statement | Data sharing plans for the current study are unknown and will be made available at a later time |
